# Networks of respiratory‐muscular coupling in exercise and fatigue in young adults

**DOI:** 10.14814/phy2.70994

**Published:** 2026-07-01

**Authors:** Sergi Garcia‐Retortillo, Javier O. Pinzon Arenas, Óscar Abenza, Hugo F. Posada‐Quintero, Plamen Ch. Ivanov

**Affiliations:** ^1^ College of Nursing University of Central Florida Orlando Florida USA; ^2^ Biomedical Engineering University of Connecticut Storrs Connecticut USA; ^3^ Faculty of Medicine and Health Sciences University of Barcelona Barcelona Spain; ^4^ Keck Laboratory for Network Physiology, Department of Physics Boston University Boston Massachusetts USA; ^5^ Department of Neurosurgery Boston University Chobanian & Avedisian School of Medicine Boston Massachusetts USA; ^6^ Institute of Biophysics and Biomedical Engineering Bulgarian Academy of Sciences Sofia Bulgaria

**Keywords:** breathing rate, complex systems, dynamic networks, electromyography, network physiology

## Abstract

Respiratory and muscular systems must integrate ventilation, oxygen delivery, and muscle activation to meet exercise demands. While decades of research have provided understanding of how these systems function individually, the principles regulating their dynamic coupling as a network remain unexplored. Our goal was to investigate how respiratory dynamics synchronize and integrate as a network with the activity of muscles during exercise, and assess how it responds to fatigue. Nine adults performed two graded cycling tests until exhaustion, starting at 0 W with 25 W·min^−1^ increments. Continuous synchronous recordings included electromyography (EMG) from right and left vastus lateralis and erector spinae, and respiration waveform via chest belt. Respiratory‐muscular coupling was measured using the amplitude–amplitude cross‐frequency coupling (ACFC) method. First, breathing rate was extracted from the respiration waveform. Second, EMG signals were decomposed into ten frequency bands [F1–F10], representing distinct neuromuscular processes. Last, cross‐correlation coefficients (C) were computed as the ACFC outcome. We uncover novel network maps of respiratory‐muscular dynamic interactions. We find that respiratory‐muscular networks exhibit a complex hierarchical structure which depends on the role muscles play during exercise. Further, cross‐correlations significantly increase with fatigue accumulation during exercise, indicating stronger integration between breathing and muscle activation under rising metabolic demand. This network‐level adaptation shows that physiological responses to exercise arise not only from isolated systems, but also from their dynamic interactions as an integrated network. The network physiology approach utilized here contributes to the development of a new class of network‐based markers to quantify multisystem interactions underlying human function during exercise.

## Graphical abstract

Amplitude‐amplitude cross‐frequency coupling (ACFC) is a novel method to quantify respiratory‐muscular dynamic interactions using breathing‐rate time series and EMG frequency‐band activity from distinct muscles. ACFC‐derived network maps show that respiratory‐muscular coupling increases with fatigue accumulation.

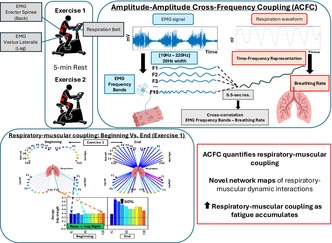

## 1 INTRODUCTION

A fundamental question in respiratory and muscle physiology is how the respiratory system operates in synchrony with skeletal muscles to maintain homeostasis, facilitate movement, and adapt to exercise demands and fatigue. While decades of research have produced a rich and comprehensive understanding of respiratory and muscular function as individual systems (Hall, 2016; Kenney et al., 2015; Sharples et al., 2022), respiratory‐muscular coupling during exercise remains poorly characterized. In particular, little is known about the dynamic patterns of coupling and network communication between the respiratory system and skeletal muscles, and how these interactions reorganize with exercise and fatigue. Addressing this basic question is essential for defining how respiratory and muscular systems interact to support physiological function and adapt to exercise and fatigue.

The respiratory and muscular systems are tightly interdependent, as respiration supplies the oxygen required by skeletal muscles to sustain ATP production while eliminating carbon dioxide produced as a byproduct of metabolism (Hall, 2016). During exercise, the increased energetic demands of contracting muscles drive a parallel rise in pulmonary ventilation to balance oxygen delivery with utilization and to maintain acid–base status (Binder et al., 2008; McArdle et al., 2015; Skinner & Mclellan, 1980). This relationship is inherently bidirectional: while muscles depend on respiration for energy, their contractions also impose both mechanical and metabolic demands that influence breathing (Gandevia, 2001). Traditional physiological studies have long documented this interdependence through measures such as maximal oxygen uptake and related pulmonary parameters (Beltz et al., 2016; Edvardsen et al., 2014; Ross et al., 2016), muscle oxygen saturation (Bangsbo & Hellsten, 1998; Casey & Joyner, 2011), blood lactate accumulation, arterial blood gases (Binder et al., 2008; Keir et al., 2022), and others. These measures reflect how closely respiration and muscle activity are linked, particularly during exercise. However, while they provide key valuable insights into individual system function, they cannot provide direct measurement on the coordinated activity between respiration and skeletal muscles (Balagué et al., 2016; Garcia‐Retortillo et al., 2017, 2019).

Studies in recent years have investigated ventilatory‐locomotor synchronization during rhythmic exercise, typically examining entrainment between breathing and locomotor rhythms (e.g., stable breath‐to‐cadence ratios or phase locking between respiratory cycles and pedal revolutions) (Daley et al., 2013; Fulton et al., 2018; Stickford et al., 2015). Such work has provided valuable insight into how respiratory timing may align with locomotor movements (e.g., gate steps). However, these approaches do not quantify multiscale dynamic coupling between respiration and dynamic measures of neuromuscular function (Ashkenazy et al., 2001). First, ventilatory‐locomotor synchronization studies generally relate respiratory variables to locomotor events (e.g., steps, cadence, accelerometry) rather than to direct measures of muscle activity (Daley et al., 2013; Fulton et al., 2018; Stickford et al., 2015). Thus, in prior studies the muscular component is inferred indirectly through number of movement events per respiratory cycle. For a more direct characterization of respiratory‐muscular coupling, direct electrophysiological measures such as surface electromyography (EMG) are required to quantify dynamics in myoelectrical activation. Second, skeletal muscle function is not a single process but reflects a heterogeneous mixture of motor‐unit and frequency‐specific muscle components that change with workload and fatigue (Garcia‐Retortillo et al., 2020; Hug et al., 2023). Accordingly, assessing how respiratory dynamics coordinate with activation in distinct EMG frequency bands across muscles is necessary to capture this complexity. To date, quantitative frameworks to assess continuous, multiscale coordination between respiratory dynamics and direct measures of muscle activity remain limited. Addressing this gap requires moving beyond isolated variables toward a dynamic and adaptive network‐based approach capable of quantifying multisystem coordination. The field of Network Physiology (Bartsch et al., 2015; Bashan et al., 2012; Ivanov, 2021; Ivanov & Bartsch, 2014) and specifically Network Physiology of Exercise (Balagué et al., 2020; Balagué, Garcia‐Retortillo, et al., 2022; Balagué, Hristovski, et al., 2022), provide the necessary theoretical framework and methods to address this fundamental question.

Approaches in physiology based on adaptive networks are an emerging framework that has only recently begun to shed light on how physiological systems coordinate their activity. Previous studies have provided insights into how the respiratory system interacts with the cardiovascular system, and how skeletal muscles coordinate both with one another and with the cardiovascular system. For example, cardio‐respiratory coupling has been examined through respiratory sinus arrhythmia and cardio‐respiratory phase synchronization (CRPS) (Angelone & Coulter, 1964; Bartsch et al., 2012, 2014; Bartsch & Ivanov, 2014; Schäfer et al., 1998; Song & Lehrer, 2003; Xu et al., 2006), revealing rhythmic and transient coordination between heartbeats and breathing cycles that depend on physiological states. Complementary approaches such as principal component analysis (PCA) further demonstrate how cardiovascular and respiratory variables dynamically coordinate to optimize oxygen transport and metabolic regulation (Abenza, Montull, et al., 2025; Garcia‐Retortillo et al., 2017, 2019; Żebrowska et al., 2020). Likewise, the concepts of cortico‐muscular and cardio‐muscular coupling have been introduced to investigate dynamic interactions between brain and muscle, as well as cardiac and muscle activity. Recent studies demonstrated network coordination among cortical and muscular rhythms across the sleep–wake cycle (Rizzo et al., 2020, 2022, 2023), and that autonomic regulation of the heart rate synchronizes with distinct myoelectrical rhythms to facilitate movement and adapt to exercise and fatigue (Langan, 2025; Garcia‐Retortillo & Ivanov, 2025). In parallel, inter‐muscular coupling has also been largely investigated, showing how muscles synchronize their activity to produce efficient movement patterns (d'Avella & Bizzi, 2005; Hug et al., 2021; Kerkman et al., 2018, 2020) and reorganize in response to exercise, fatigue (Abenza, Garcia‐Retortillo, et al., 2025; Boonstra et al., 2008; Garcia‐Retortillo et al., 2023; Garcia‐Retortillo & Ivanov, 2022; Kattla & Lowery, 2010), or aging (Garcia‐Retortillo, Abenza, Thiamwong, et al., 2025; Garcia‐Retortillo, Abenza, Vasileva, et al., 2025; Garcia‐Retortillo et al., 2020). Collectively, these works highlight the integrative nature of physiological function and the promise of a dynamic and adaptive network‐based approach. Yet, despite the clear physiological interdependence between the respiratory and skeletomuscular systems, the basic principles regulating respiratory‐muscular coupling remain not understood.

Previous studies have shown that cardiac and respiratory dynamics are integrated across multiple temporal scales, exhibiting power‐law long‐range correlations that reflect the hierarchical organization of autonomic control (Ivanov et al., 2004, 2009; Schumann et al., 2010). Moreover, cardio‐muscular coupling analyses revealed that cardiac oscillations are embedded within myoelectrical activity (Garcia‐Retortillo & Ivanov, 2025), suggesting that shared cardiac regulatory mechanisms coordinate with the activity of distant muscles. By analogy, we first hypothesized the existence of a network of coordinated interactions between respiratory dynamics and muscle activity, exhibiting a hierarchical organization of coupling strength. Second, we hypothesized that coupling strength within this respiratory‐muscular network would depend on muscle type and functional role during exercise (e.g. major, supportive). Third, we hypothesized that the degree of respiratory‐muscular coupling would change and adapt in response to exercise demands, as central neural drive to the respiratory system increases and neuromuscular fatigue accumulates.

To establish first building blocks of the respiratory‐muscular network interactions, we apply the amplitude‐amplitude cross‐frequency coupling (ACFC) method (Abenza, Garcia‐Retortillo, et al., 2025; Garcia‐Retortillo, Abenza, Thiamwong, et al., 2025; Garcia‐Retortillo, Abenza, Vasileva, et al., 2025; Garcia‐Retortillo et al., 2023; Garcia‐Retortillo & Ivanov, 2025; Garcia‐Retortillo & Ivanov, 2022), which quantifies the co‐modulation between fluctuations in breathing rate and EMG spectral power activation in distinct frequency bands across a range of temporal scales. The ACFC method enables identification of patterns of coupling and network interactions between breathing dynamics and distinct myoelectrical rhythms across muscles. Further, the method quantifies how these network interactions evolve over time in response to exercise and fatigue. Unlike phase‐locking approaches used in earlier ventilatory‐locomotor synchronization studies (Daley et al., 2013; Fulton et al., 2018; Stickford et al., 2015), which focus on short time scale breath‐to‐step timing relationships, ACFC captures coordinated temporal fluctuations in signals' amplitude over multiple scales and their evolution in time. Accordingly, ACFC can characterize features of respiratory‐muscular coupling during exercise that cannot be detected using a strict breath‐to‐cadence entrainment framework.

Our goal was to investigate how respiratory dynamics integrates with the activity of distinct muscles as a network during two consecutive cycling tests performed until exhaustion, and examined how the network of respiratory‐muscular interactions reorganizes with fatigue. We uncovered that the respiratory‐muscular network exhibits a complex hierarchical organization of distinct sub‐networks and network modules, which depend on the role muscles play during the cycling movement and histochemical composition. Our findings show that the respiratory‐muscular network dynamically reorganizes in response to fatigue to meet the increasing demands of exercise. The framework used in this study opens new avenues for developing network‐based markers with the potential to characterize respiratory‐muscular, inter‐muscular, and muscle‐organ interactions during exercise, as well as to assess fatigue levels, fitness status, and the effectiveness of cardiorespiratory and muscular training programs.

## 2 MATERIALS AND METHODS

### 2.1 Participants

To determine the sample size for this study, a power analysis was conducted using G‐Power 3.1 (Faul et al., 2007) using the Wilcoxon signed‐rank test for matched pairs. Previous studies using a comparable within‐subject design (two repeated cycling tests to exhaustion) reported large fatigue‐related effects (≈1 or higher (Garcia‐Retortillo et al., 2017)). Assuming an effect size = 1.0, *α* = 0.05, and power (1 − *β*) = 0.80, the estimated minimum required sample size was *n* = 9 participants. Nine young adults (six females and three males; age 21.22 ± 1.09 years, height 157.48 ± 9.14 cm and mean body mass 65.37 ± 9.62 kg) were recruited for this study. Participants were strictly recruited according to the following inclusion criteria: (a) aged 18–30 years; (b) body mass index (in kg/m2) >18.5 and <30; (c) normal physical activity >5 and <10 h/week, but without sport specialization (not active athletes); and (d) blood pressure <140/90 mmHg. The following exclusion criteria were applied: (a) intake of prescribed drugs that could negatively affect muscle strength, such as corticosteroids; (b) no current or previous injury that could prevent performance during the experimental protocol test; and (c) any other physical condition (cardiac, respiratory, etc.) that might have prevented the performance of a test protocol involving cycling exercise until exhaustion. The experimental protocol was approved by the local ethical committee (Wake Forest University IRB00024843) and was carried out according to the Declaration of Helsinki. Before taking part in the study, participants read the study description and risks and gave informed consent in writing. All ethical regulations were followed.

### 2.2 Study design and experimental protocol

After providing written informed consent, participants completed a single laboratory visit. During this session, they performed two consecutive graded cycling tests (Cosmed E100 ergometer) to exhaustion. The first test (Exercise 1) started at 0 W, with workload increasing by 25 W per minute until the participant could no longer maintain the prescribed cadence of 70 rpm for more than 5 consecutive seconds. Participants were instructed to keep the trunk upright, maintain relaxed shoulders, and focus primarily on leg movement to minimize motion artifacts in the recorded signals. After a 5‐min rest period, participants completed a second cycling test (Exercise 2) following the same protocol.

### 2.3 Respiration and electromyography acquisition and signal processing

Participants were asked to wear appropriate clothing that would not obstruct EMG electrode placement sites. Before the mounting of the respiration belt and EMG electrodes, participants' skin was shaved and cleaned using alcohol and left to dry for 60 s to reduce the myoelectrical impedance, according to the SENIAM guidelines (Hermens et al., 2000).

The respiration waveform was measured using a belt equipped with a precision transducer to detect changes in thoracic circumference. The belt was securely positioned around the upper thorax, approximately at the level of the axillae. The respiration belt provided the most appropriate compromise between physiological relevance, temporal resolution, and methodological compatibility with the analytical method used in this study. Our objective is to quantify respiratory‐muscular interactions using the amplitude‐amplitude cross‐frequency coupling (ACFC), a method that requires continuous, uniformly sampled, high‐resolution, and synchronized time series across physiological systems. In the present analysis, breathing frequency (BR) was selected as the primary respiratory variable because it reflects rapid adjustments in respiratory timing during exercise and is closely modulated by central command and afferent feedback from the working muscles (group III and IV afferents). In contrast, other relevant ventilatory variables such as tidal volume exhibit different regulatory behavior during exercise and are typically measured on a breath‐by‐breath basis (Keir et al., 2014), which introduces two methodological limitations for the present analysis. First, breath‐by‐breath data typically yield one value per respiratory cycle, resulting in substantially lower temporal resolution compared to the continuous respiration waveform and EMG recordings. Second, and most importantly, breath‐by‐breath data are inherently event‐based and therefore irregularly sampled; as breathing rate increases during exercise, the temporal spacing between data points changes. This variable sampling structure complicates synchronization with uniformly sampled high‐frequency EMG signals and limits the applicability of ACFC. Although alternative systems may provide higher temporal resolution than breath‐by‐breath measurements, they remain limited for the present analysis and are typically acquired in separate systems, which may constrain precise synchronization with EMG recordings.

EMG signals were also recorded simultaneously with the respiration waveform from the following muscles: left and right vastus lateralis (Leg‐Left and Leg‐Right muscles); left and right erector spinae longissimus (Back‐Left and Back‐Right lower back muscles). The exact location of the surface electrodes' (Meditrace Foam EG200, Danlee Medical Products Inc., Syracuse, USA) placement on each muscle was carried out according to the recommendations of the SENIAM organization. More specifically, vastus lateralis electrodes were placed two thirds of the way along the line from the anterior spina iliaca superior to the lateral side of the patella, and the erector spinae electrodes were located a two‐finger width lateral from the spinous process of vertebra L1. Muscle selection was guided by both functional relevance and methodological consistency. Specifically, we focused on bilateral lower limb muscles (vastus lateralis) that serve as primary movers during cycling, as well as the erector spinae muscles, which play a key postural role in stabilizing the trunk (Duc et al., 2008; Turpin et al., 2016). After the electrodes were secured with tape to minimize movement artifacts, a signal quality check was performed to ensure respiration and EMG signal validity. The respiration belt and electrodes were removed after completing Exercise 2 (i.e., second cycling test). A Biopac MP150 unit (Biopac Systems Inc., Goleta, CA, USA) was used to collect synchronized respiration waveform (BN‐RSPEC2‐T) and EMG (BN‐EMG2‐T) signals.

Data was processed using MATLAB (MathWorks, Natick, MA, USA). Raw respiration and EMG signals were recorded at a sampling frequency of 2000 Hz. The respiration waveform was filtered with a 2 Hz low‐pass and 0.1 Hz high‐pass Butterworth filter to isolate the relevant breathing frequencies. Typically, a healthy individual at rest breathes approximately 12 times per minute; during moderate exercise, this rate increases to 20–40 breaths per minute and can reach up to ~60 breaths per minute at near‐maximal effort. Thus, the BR during exercise generally ranges between 0.1 and 1 Hz (Nicolò et al., 2017, 2018, 2020). EMG data were filtered using a 5–250 Hz bandpass filter. A Notch filter was used with a width of 1 Hz at the frequency of 60 Hz to remove line interference from the grid.

### 2.4 Amplitude‐amplitude cross‐frequency coupling (ACFC) analyses

To quantify respiratory‐muscular coordination, we used the amplitude‐amplitude cross‐frequency coupling method (ACFC), that has been recently developed and validated to assess multisystem coordination (Abenza, Garcia‐Retortillo, et al., 2025; Garcia‐Retortillo, Abenza, Thiamwong, et al., 2025; Garcia‐Retortillo et al., 2020, 2023; Garcia‐Retortillo & Ivanov, 2025; Garcia‐Retortillo & Ivanov, 2022).

#### 2.4.1 Instantaneous breathing rate time series

**FIGURE 1:**
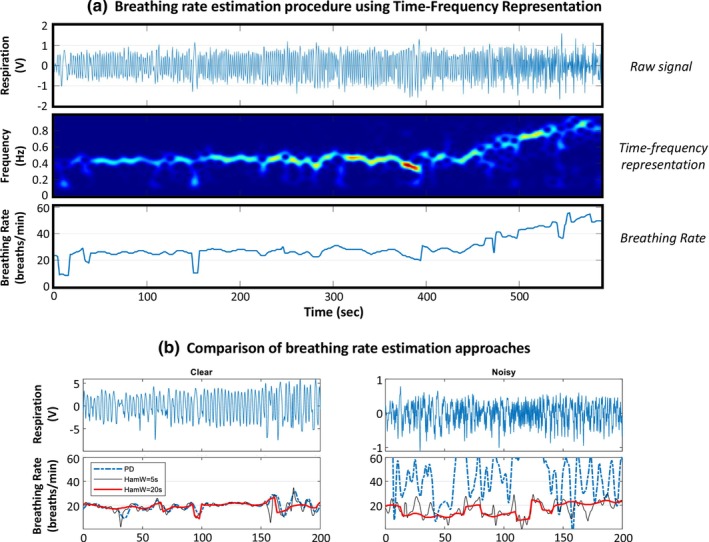
Instantaneous breathing rate time series. (a) To estimate the BR, the TFS of the filtered respiration signal is computed, and then the frequency with the maximum power at each time step is extracted (blue and red colors refer to low and high spectral power, respectively). Finally, frequency is multiplied by 60, and a move‐mean average filter is passed to smooth the estimated BR. (b) Comparison of BR time series estimation using PD and TFR with 5‐ and 20‐s windows for clear and noisy respiration waveforms. When the waveform was clean, all the approaches showed similar BR estimation. However, when the waveform is was noisy, PD detected false respiration cycles, estimating large BR with high variability, while TFR could gather the actual respiration cycle frequency. The larger the TFR analysis window, the smoother BR is estimated.

We tested two approaches to estimate the BR time series for both Exercise 1 and Exercise 2. For the first approach, we used breath peak detection (PD). This consisted of downsampling the respiratory waveform and detecting the maximum values of the segments that were above a threshold. Next, we multiplied the inverse of the obtained inter‐breath peak intervals by 60 to calculate the BR in breaths per minute (bpm). Finally, a cubic spline interpolation was performed to generate a BR time series sampled at 2 Hz. For the second approach, we used a time‐frequency representation (TFR) of the respiratory waveform (Auger et al., n.d.). TFR is a powerful tool for extracting instantaneous frequencies from different types of physiological signals, enabling the combination of time‐ and frequency‐domain analyses (Iatsenko et al., 2016). First, the respiration waveform was downsampled to 2 Hz. Then, the power spectral density of the signal was calculated using a sliding window. Next, we obtained the frequency with the highest spectral power at each time point and multiplied it by 60 to obtain the BR time series in bpm. Since this technique uses analysis windows to obtain spectral power, selecting a suitable window size is necessary. For BR estimation, a five‐second window size is commonly used. However, short windows are sensitive to noise, which makes BR highly variable. Multiple studies have used longer windows of over 30 s, which provide a smoother BR calculation (Charlton et al., 2018; Chin et al., 2024). We explored two window lengths: the default five‐second length and a 20‐s length. Our final choice was a 20‐s window (which includes at least four respiratory cycles) with 0.5 s overlap, producing smoother BR time series with 0.5 s resolution. Last, we applied a two‐second moving average filter to the BR time series. Figure 1a shows an example of the respiration waveform, the TFR spectrogram, and the estimated BR for a representative participant.

#### 2.4.2 Spectral decomposition of EMG signals: EMG frequency band time series

**FIGURE 2:**
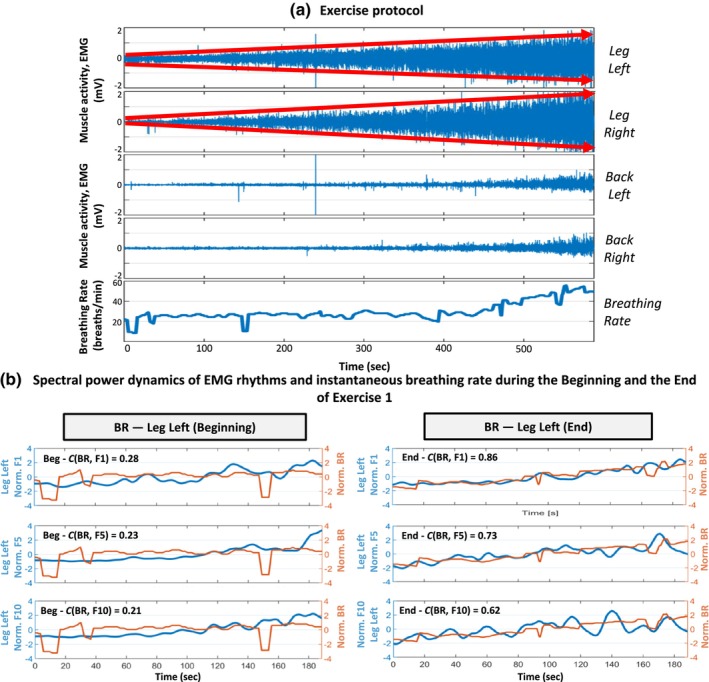
Dynamics of breathing rate and spectral power of EMG frequency bands from one representative participant. (a) Breathing rate (BR) time series and EMG signals recorded from each muscle. Both BR and EMG amplitude gradually increase with accumulation of fatigue during the cycling test performed until exhaustion. (b) Normalized time series of BR (Orange lines; right vertical axis) and spectral power EMG frequency bands (F1, F5, and F10; blue lines; left vertical axis) in the Beginning (left panels) and End (right panels) segments of Exercise 1 for the Respiration‐LegL sub‐network. At the Beginning of Exercise 1, the EMG spectral power time series were weakly coupled with BR. In contrast, at the End of Exercise 1, coupling strength increased markedly across all frequency bands, as reflected by higher correlation coefficients C between BR and the EMG frequency bands. By the end of exercise, BR shows a gradual rise and becomes less variable due to parasympathetic withdraw, while EMG frequency‐band time series progressively increase, indicating greater motor‐unit recruitment and overall neuromuscular activation with fatigue accumulation.

EMG signals were divided into 20‐s window with a 0.5‐s step across the entire duration of Exercise 1 and Exercise 2, separately. This long window length is essential because respiration is a low‐frequency signal, and windows of at least 20 s are required to encompass multiple respiratory cycles and to extract meaningful cross‐frequency coupling components. Within each 20‐s time window: (i) the spectral power $\tilde{S}$_*δ*(*f*)_(*t*_*i*_) was extracted from each EMG signal (based on the discrete Fourier transform), and (ii) $\tilde{S}$_*δ*(*f*)_(*t*_*i*_) values were computed in bins of 0.5 Hz within the range 10–250 Hz, resulting in *N* = 480 data points for each window. To calculate the spectral power within each 20‐s time windows, we used the ‘*pwelch*’ function in MATLAB, which computes the power spectral density of the EMG signal through Welch's method. Spectral power values were obtained in each 20‐s window with step of 0.5‐s, thus, producing a time series with 0.5‐s resolution for each predefined frequency band [F1,…,F10] (see detailed description in (Abenza, Garcia‐Retortillo, et al., 2025; Garcia‐Retortillo, Abenza, Vasileva, et al., 2025; Garcia‐Retortillo et al., 2023; Garcia‐Retortillo & Ivanov, 2025; Garcia‐Retortillo & Ivanov, 2022)) (Figure 2). The final 0.5‐s resolution of the EMG frequency band time series matches the 0.5‐s resolution of the BR.

To examine the contribution from different frequency bands *F*_*i*_ to the EMG spectral power within each 20‐s window, we defined 10 frequency bands with equal width of 19.5 Hz: F1 = [10–29.5 Hz], F2 = [30–49.5 Hz], F3 = [65–84.5 Hz], F4 = [85–104.5 Hz], F5 = [105–124.5 Hz], F6 = [125–144.5 Hz], F7 = [145–164.5 Hz], F8 = [165–184.5 Hz], F9 = [185–204.5] and F10 = [205–224.5 Hz]. Notably, the relationship between EMG spectral power frequency bands and muscle fiber types is not straightforward, as spectral power is influenced by various factors such as motor unit synchronization, tissue filtering effects, and electrode placement (Del Vecchio et al., 2017; Farina, 2008; von Tscharner & Nigg, 2008). Nevertheless, lower‐frequency components (<30–40 Hz; F1–F2) are consistent with motor units exhibiting lower muscle fiber conduction velocities and longer‐duration motor unit action potentials (MUAPs), features typically associated with low‐threshold motor units, which are often composed predominantly of slow‐twitch fibers. In contrast, recruitment of higher‐threshold motor units—generally characterized by larger axon diameters, higher conduction velocities, and shorter‐duration MUAPs—broadens the EMG spectrum toward higher frequencies. (Dreibati et al., 2010; Jones et al., 1979; Kesar & Binder‐Macleod, 2006). Higher frequencies (above ~100 Hz) primarily reflect MUAP waveform characteristics rather than motor unit discharge rate per se. MUAPs consists of short‐duration electrical events (approximately 5–10 ms) that contribute to the high‐frequency content of the EMG signal due to: the steep rising and falling phases of MUAP waveforms (shape), the temporal summation/overlap of multiple MUAPs, and muscle fiber conduction properties (Merletti & Farina, 2016; Prilutsky, 2000). Fast‐twitch muscle fibers, which generate shorter‐duration action potentials, may contribute more significantly to this higher‐frequency component, extending the EMG spectral content into the 100–230 Hz range. Importantly, the defined frequency bands should not be interpreted as exclusive markers of specific fiber types, but rather as reflecting shifts in the relative contribution of motor units with differing electrophysiological properties.

Next, the sum of the power $\tilde{S}\left(f\right)$ across all frequency bins (0.5 Hz width) within each frequency band (19.5 Hz width) was calculated as $\tilde{S}\left(f\right)$: = $\sum_{i=1}^{n}S\left(f_{i}\right)$, where *ƒ*_*i*_ represents all *n* = 39 frequency bins in each frequency band *F*_*i*_. This yielded ten time series of EMG band power $\tilde{S}$_*δ(f)*_(*t*_*i*_), each with 0.5‐s resolution for each muscle, representing the dynamics of EMG activity rhythms.

#### 2.4.3 Cross‐correlations between time series of instantaneous breathing rate and EMG frequency bands

**FIGURE 3:**
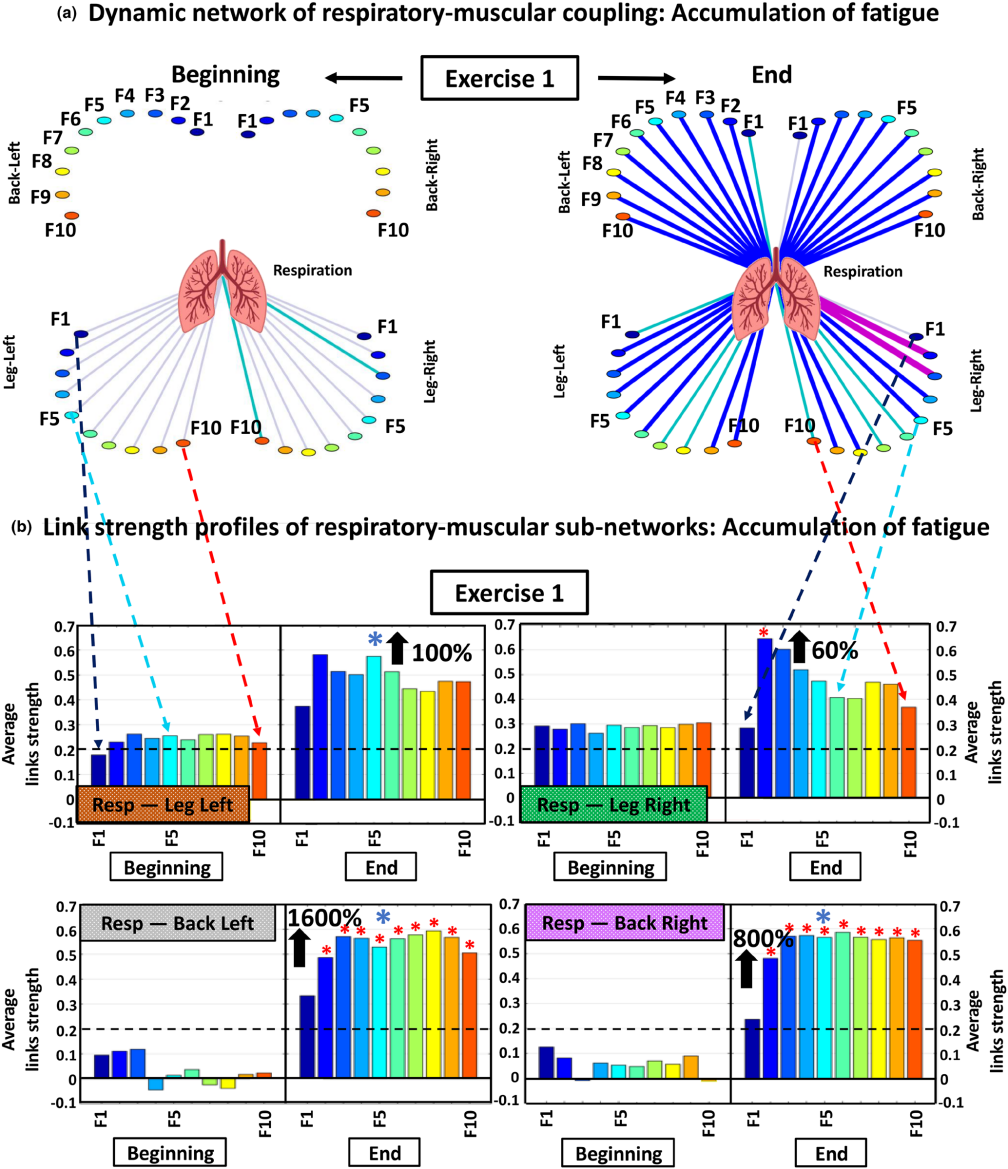
Dynamic network of respiratory‐muscular coupling, and its reorganization with fatigue accumulation. (a) Dynamic network of respiratory‐muscular interactions between respiration and major muscles involved in the cycling test of Exercise 1. Group‐averaged map where network links represent the links strength (correlation coefficients *C*(BR, *F*_*i*_)) between breathing rate (BR) and myoelectrical rhythms (frequency bands *F*_*i*_) from Leg and Back muscles. Each respiratory‐muscle sub‐network contains 10 links quantifying the degree of coupling between BR and 10 muscle frequency bands *F*_*i*_. Line width and color indicate link strength (thicker/darker lines correspond to stronger coupling, Methods). Network maps derived from data corresponding to the Beginning (first third) and End (last third) segments of Exercise 1 show reorganization of the respiratory‐muscular network with accumulation of fatigue. Each muscle is represented as a semicircle with color nodes for different frequency bands *F*_*i*_. Coupling between BR and EMG frequency bands from LegL/LegR and BackL/BackR muscles from a dynamic multiplex network with pronounced heterogeneity, characterized by distinct topology and a hierarchical organization of link strength. (b) Links strength profiles of respiratory‐muscular sub‐networks. Each of the 10 bars in the profile represents a link in the corresponding sub‐network in (a), and quantifies the coupling strength *C*(BR, *F*_*i*_) between BR and the EMG frequency bands *F*_*i*_ from a given muscle during Exercise 1. With fatigue accumulation at the End of Exercise 1, a significant increase in links strength between BR and EMG frequency bands were observed across sub‐networks. The magnitude of this increase differed between Respiration‐Leg and Respiration‐Back sub‐networks (∼60%–100% vs. ∼800%–1600%, respectively). At the End of Exercise 1, the strongest coupling in the Respiration‐Leg sub‐network occurred in the (30–50 Hz) band, typically associated with common corticospinal inputs. In the Respiration‐Back sub‐networks, links strength was weak for lower frequency bands [F1‐F2] but increased across the higher frequency bands [F3‐F10] (65–225 Hz). Blue stars indicate statistically significant differences between the Beginning and End when link strength is averaged across all frequency bands. Red stars indicate statistically significant Beginning–End differences for each individual frequency band (Wilcoxon signed‐rank test, *p* < 0.05).

**FIGURE 4:**
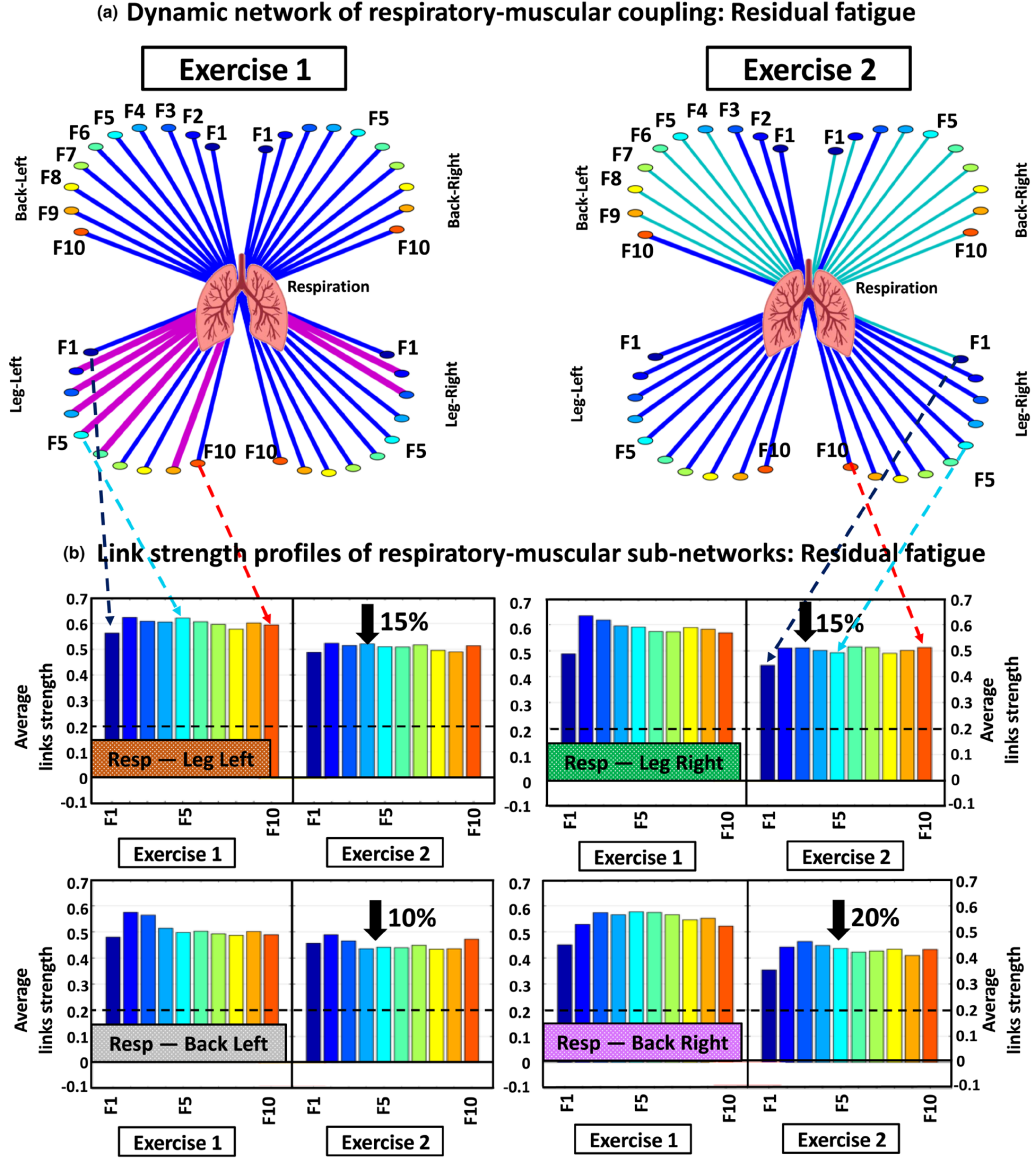
Dynamic network of respiratory‐muscular coupling, and network reorganization with residual fatigue across exercise bouts. (a) Dynamic networks of respiratory‐muscular coupling between breathing rate (BR) and EMG frequency bands from Leg/Back muscles involved in the cycling test for consecutive Exercise 1 and Exercise 2 bouts. Shown are group‐averaged network maps, where network links represent the coupling (correlation coefficients *C*(BR, *F*_*i*_)) between BR and myoelectrical rhythms (frequency bands *F*_*i*_) from Leg and Back muscles. Sub‐networks in the respiratory‐muscular network contain 10 links that quantify the degree of coupling between BR and 10 bands *F*_*i*_ within a given muscle. Each muscle is represented as semicircle with color nodes for different bands *F*_*i*_. Line width and color indicate network link strength (thicker/darker lines correspond to stronger coupling, Methods). Network maps derived from data during Exercise 1 and Exercise 2 show reorganization of topology and link strength with residual fatigue. (b) Links strength profiles of respiratory‐muscular sub‐networks for consecutive exercise bouts. Each of the 10 bars in the profile represents a link in the corresponding sub‐network in (a), and quantifies the coupling through correlation coefficients *C*(BR, *F*_*i*_) between BR and the EMG frequency bands *F*_*i*_ from a given muscle. Comparing Exercise 2 to Exercise 1, a ∼10%–20% decline in link strength was observed across all respiratory‐muscular sub‐networks, reflecting residual fatigue from Exercise 1, with partial but incomplete recovery of respiratory‐muscular coupling. Despite this reduction, the overall shape of the links strength profiles remained consistent across frequency bands and across all sub‐networks.

Notably, the analyses described in this section were performed separately for: (i) Beginning (first third) versus End (last third) segments of Exercise 1 (Figure 3), and (ii) Exercise 1 versus Exercise 2 (Figure 4). We considered all possible pairs between respiration and selected muscles (i.e. respiration–muscle sub‐networks) involved in the cycling test (Respiration‐LegL; Respiration‐LegR; Respiration‐BackL, and Respiration‐BackR).

For each respiration–muscle sub‐network, we computed the bivariate equal‐time Pearson's cross‐correlation between BR and all EMG spectral power frequency bands *F*_*i*_ where *i* = 1,…,10. This led to 1 × 10 = 10 cross‐correlation values C for each respiration–muscle sub‐network, as shown in the bar plots in Figures 3b and 4b. Importantly, for the Beginning vs. End comparison (Figure 3), BR and EMG spectral power frequency band time series were normalized separately within each segment to a zero mean (*μ* = 0) and a unit standard deviation (*σ* = 1) before computing the cross‐correlation. In contrast, for the Exercise 1 vs. Exercise 2 comparison (Figure 4), they were normalized over the entire test duration. These normalized time series reflect the micro‐scale (0.5‐s resolution) of synchronous modulation in the amplitude of muscle activation, and enable to track variations in network interactions between EMG frequency bands *F*_*i*_ throughout the entire exercise. Note that C quantifies the degree of coupling between BR and the EMG frequency band *F*_*i*_ from one muscle. The cross‐correlation values ranged from C = −1 (fully anti‐correlated) to C = 1 (fully positively correlated), with C = 0 indicating the absence of linear relation between BR and the power time series of a given EMG frequency band. When cross‐correlating the BR time series with the spectral power time series of one EMG frequency band for an entire exercise bout, the strongest coefficients consistently occurred at zero lag (i.e., no delay).

#### 2.4.4 Network maps of respiratory‐muscular coupling

A multiplex network of sub‐networks was obtained to visualize interactions from all respiration–muscle pairs and their hierarchical organization within the network (Figures 3a and 4a). We mapped the results of our cross‐correlation analyses (Section 2.4.3) into different group‐averaged networks for the Beginning (first third) and End (last third) segments of the test (Figure 3a), and for the entire Exercise 1 and 2 (Figure 4a). This graphical representation we employed is essential for identifying patterns in the respiratory‐muscular network structure, visualizing the hierarchical organization of sub‐networks and modules, and tracking the transition in network characteristics with the accumulation of fatigue. Each muscle is represented by a semicircle where colored nodes represent distinct frequency bands *F*_*i*_, representing distinct neuromuscular processes. Network links correspond to the cross‐correlation values C obtained from the cross‐correlation analysis (Section 2.4.3) and reflect the coupling strength between BR and a given frequency band from the muscle in the respiration–muscle sub‐network. Link strength is marked by line color and width—we use a distinct link color code to demonstrate network reorganization with fatigue: 0.15 <Ci,j <0.3 (very thin gray lines), intermediate links (0.3 <Ci,j <0.45; thin green lines), strong links (0.45 <Ci,j <0.6; dark blue thick lines) and very strong links (Ci,j >0.6; magenta very thick lines). Links corresponding to cross‐correlation values Ci,j <0.15 are not shown on the network maps.

#### 2.4.5 Additional control analyses: Trends, surrogate tests, and physiological threshold

To assess the relative contribution of the trends in physiological signals to the cross‐correlation measures and derived respiratory‐muscular networks, we performed additional analyses in which both BR and EMG spectral power time series were detrended prior to cross‐correlation computation.

Next, we performed a Fourier phase‐randomization surrogate analysis on the EMG recordings (LegL, LegR, BackL, BackR) to assess whether the observed respiratory‐muscular coupling could arise from shared spectral structure or protocol‐driven trends alone (Kantz & Schreiber, 2003; Schreiber & Schmitz, 2000; Theiler et al., 1992). This method preserves the amplitude distribution of the original EMG signal and its power spectrum across frequency bands *F*_*i*_, while randomizing the Fourier phases. As a result, the surrogate EMG signals retain the global spectral power content and the relative distribution of power across frequency bands, but their fine temporal organization and phase‐dependent temporal structure are disrupted. Importantly, this surrogate assesses whether the observed respiratory‐muscular correlations depend on preserved temporal organization rather than on shared marginal spectral properties or slow monotonic trends. After generating the surrogate EMG signals, we recomputed EMG spectral power time series using the same 20‐s windowing procedure and repeated the cross‐correlation analysis with the original BR time series, following the same method used for the original data.

Finally, to assess the statistical baseline and interpretational reference of the observed network organization and its changes with fatigue, we introduced an additional step in our surrogate test to determine the significance threshold of this dataset for network links strength. Specifically, for each network link, we generated surrogates cross‐correlating signals from all possible pairs of randomly chosen subjects. Since we have 9 participants in the database, 36 pairs of random subject combinations were generated. Each respiration‐muscle pair (Respiration‐LegL, Respiration‐LegR, Respiration‐BackL, and Respiration‐BackR) involves 10 links between BR and the 10 EMG frequency bands *F*_*i*_ of the corresponding muscle in the pair. Thus, combining the four sub‐networks representing all respiration‐muscle pairs we obtained a distribution of 1440 surrogate links (cross‐correlation values) for Exercise 1 and Exercise 2—that is, 4 respiration–muscle pairs × 10 links × 36 subject combinations = 1440 surrogate links. For each exercise distribution, the mean μsurr and standard deviation σsurr were obtained. Thus, the physiological significance threshold for Exercise 1 and Exercise 2 at a 95% confidence level for the network link strength was defined as μsurr +2σsurr.

### 2.5 Statistical tests

Since the statistical analysis was done on data belonging to measurements at two times periods of the same subjects, and the respiratory‐muscular coupling was not normally distributed, Wilcoxon's matched‐pairs test (significance level of 5%) was employed. Additionally, due to multiple comparisons on coupling strength across multiple frequency bands, alpha correction was applied using false discovery rate (FDR) correction with the Benjamini‐Hochberg method to control the proportion of false discoveries in each muscle.

## 3 RESULTS

We identified and characterized the network of respiratory‐muscular interactions during two consecutive cycling tests performed until exhaustion and assessed how the network structure reorganizes across two repeated exercise bouts. Respiration waveform was collected alongside EMG signals from four muscles: left and right vastus lateralis (LegL and LegR) and left and right erector spinae (BackL and BackR).

### 3.1 Breathing rate

Figure 1 a exemplifies the results of the TFR over a complete test. The TFR was able to estimate the respiration cycle without losing data in long breaths followed by short breaths (small in amplitude) and in continuous small breaths. For instance, at the 10‐s mark, the maximum power was below 0.2 Hz for a long breath, followed by an increase to over 0.4 Hz due to the small breath. At the 580‐s mark, many small breaths were observed. Despite the detection of many low and high frequencies, possibly due to noise, the TFR maintained an incremental BR trend over time during fatigue, computing a maximum spectral power of about 0.8 Hz. TFR avoided the influence of that noise on BR estimation.

The comparison of BR estimation using the PD and TFR approaches on clear and noisy respiration waveforms is shown in Figure 1b. In the clear waveform, the computed BR values are nearly identical. However, when noise below 2 Hz is present in the signal (e.g., due to movement during the test), the BR values obtained using the PD approach are more variable and incorrectly estimated due to incorrectly detected peaks. TFR, on the other hand, produces a less variable signal, even with a short‐time length window. Furthermore, using a larger window size mitigates rapid variations, resulting in a smoother waveform and a more precise breathing cycle frequency calculation. For this reason, we selected the BR time series obtained via TFR with a 20‐s window size.

Accumulation of fatigue during Exercise 1 was reflected by a gradual increase in the amplitude of EMG signals from both Leg and Back muscles (Figure 2a). These trends in EMG amplitude, highlighted by red lines, capture the progressive rise in myoelectrical activity. In parallel, BR exhibited a gradual rise and became less variable over time with accumulated fatigue (Bottom Panel Figure 2a). Figure 2b illustrates the degree of coupling between BR and the spectral power of different frequency bands in the Beginning (first third) and the End (last third) of Exercise 1 for a representative participant. At the Beginning of Exercise 1, the EMG spectral power time series were weakly coupled with BR. In contrast, at the End of Exercise 1, a marked increase in coupling strength was observed across all frequency bands. This increase was quantified as a marked increase in the correlation coefficients C between BR and EMG frequency bands.

### 3.2 Respiratory‐muscular networks during exercise 1: Beginning versus end

Respiratory‐muscular coupling formed a multiplex network with pronounced heterogeneity characterized by distinct topology and hierarchical structure that reorganized markedly in response to accumulated fatigue (Figure 3a). Figure 3b illustrates the link strength profiles for all respiration‐muscle sub‐networks (Resp‐LegL, Resp‐LegR, Resp‐BackL, Resp‐BackR). Each of the 10 bars in the profile represents a link in the corresponding network in Figure 3a and quantifies the degree of coupling *C*(BR, *F*_*i*_) between BR and the EMG frequency band *F*_*i*_ from one muscle.

At the Beginning of Exercise 1, Respiration‐Back sub‐networks (bottom panels Figure 3b) showed weaker link strength compared to Respiration‐Leg sub‐networks (top panels Figure 3b). Specifically, Respiration‐Leg sub‐networks displayed a relatively uniform distribution of link strength across frequency bands, whereas Respiration‐Back sub‐networks exhibited higher link strength in the lower frequency bands [F1–F3].

With fatigue accumulation at the End of Exercise 1, a significant increase in links strength between BR and EMG frequency bands was observed (Figure 3b). When averaged across frequency bands, relative large increases were observed in both leg and back muscles, with more pronounced changes in the back. Links strength increased by approximately 100% in Resp–LegL (*p* < 0.05), 60% in Resp–LegR (*p* = 0.20), 1600% in Resp‐BackL (*p* < 0.01), and 800% in Resp‐BackR sub‐network (*p* < 0.01). Specifically, at the End of Exercise 1, the weakest link strength in the Resp‐Leg sub‐networks was found in (10–30 Hz), whereas the strongest was observed in (30–50 Hz). In the Resp‐Back sub‐networks, the weakest links were in [F1‐F2], while links strength across [F3–F10] was stronger and more uniform. Red stars in Figure 3b indicate statistically significant differences comparing Beginning to End of Exercise 1 for each specific frequency band.

### 3.3 Respiratory‐muscular networks: Exercise 1 and exercise 2

Considering the entire Exercise 1 and Exercise 2, the global respiratory‐muscular network also exhibited a marked heterogeneity and hierarchical organization across sub‐networks (Figure 4a). In Exercise 1, Respiration‐Leg sub‐networks exhibited stronger link strength compared to Respiration‐Back sub‐networks (∼10%), with the strength of the links remaining uniform across all frequency bands and sub‐networks. With residual fatigue in Exercise 2, an overall decline of approximately 10–20% in link strength was observed across all sub‐networks (Resp‐LegL, Resp‐LegR, Resp‐BackL, Resp‐BackR). However, despite this reduction, no statistically significant differences were observed comparing Exercise 1 to Exercise 2 (Resp‐LegL: *p* = 0.098; Resp‐LegR: *p* = 0.074; Resp‐BackL: *p* = 0.496; Resp‐BackR: p = 0.098; Figure 4b).

### 3.4 Additional control analyses: Trends, surrogate tests, and physiological threshold

#### 3.4.1 Trends in breathing rate and EMG data

The incremental cycling protocol induced a slow incremental trend in both breathing rate (BR) and EMG spectral power time series, which reflects the structure of the experimental paradigm. In the present study, we preserved this slow component in the primary analysis for four reasons. First, while non‐stationarities and trends in the characteristics of physiological signals can influence cross‐correlation estimates (Podobnik et al., 2007, 2009), they carry very relevant physiological information about systems regulation and adaptation to perturbations such as exercise‐induced fatigue. Specifically, the progressive increase in BR and EMG spectral power reflects the fundamental physiological response to accumulating fatigue. The central aim of this study is to quantify how respiratory‐muscular coupling evolves under progressive fatigue. Removing the slow trend would remove an essential part of the physiological response to fatigue that the protocol is designed to elicit. Second, the graded cycling test is intentionally structured to induce a gradual increase in metabolic demand and neural drive. The slow‐timescale trend observed in both BR and EMG spectral power reflects this intrinsic structure of the protocol. In this context, this shared slow modulation is a physiologically meaningful outcome of the graded protocol design. Third, respiration is a low‐frequency physiological signal. Coordinated modulation between respiratory dynamics and neuromuscular activation is therefore expected to manifest over longer observation windows rather than at very short timescales. For this reason, EMG spectral power was computed using 20‐s windows, enabling detection of coordinated slow‐timescale fluctuations compatible with respiratory dynamics. Removing the slow component in signals trend could therefore obscure physiologically relevant integration occurring at longer timescales. Fourth, the magnitude of the resulting cross‐correlation depends on the similarity of the temporal trajectories of the two signals. If BR and EMG spectral power increase with parallel trends in signals amplitude, the cross‐correlation coefficient will be high. In contrast, if one signal exhibits a steeper increase relative to the other, the cross‐correlation will remain lower despite the presence of a shared upward trend. Thus, retaining the trend in our analysis allows assessment of the degree to which respiratory and myoelectrical activation scale together under progressive exercise demands.

To assess the robustness of the observed coupling to removal of this slow trend, we performed an additional analysis in which both BR and EMG spectral power time series were detrended prior to cross‐correlation computation. Following detrending, absolute correlation values decreased substantially, indicating that the slow trend contributes substantially to the overall magnitude of correlation coefficients. However, the fatigue‐dependent effect (Beginning vs. End comparison) persisted. Even after removing the slow monotonic trend, respiratory‐muscular coupling remained higher at the End compared to the Beginning of exercise (mean correlation coefficients averaged across all frequency bands; Respiration‐LegL: −0.009 ± 0.002 vs. 0.03 ± 0.02; Respiration‐LegR: 0.002 ± 0.01 vs. 0.01 ± 0.02; Respiration‐BackL: −0.03 ± 0.04 vs. 0.04 ± 0.01; Respiration‐BackR: −0.07 ± 0.03 vs. 0.02 ± 0.01).

#### 3.4.2 Surrogate test and physiological threshold

After generating the surrogate EMG signals, we recomputed EMG spectral power time series using the same 20‐s windowing procedure and repeated the cross‐correlation analysis with the original BR time series, following the same method used for the original data. Following phase randomization, correlation values were markedly reduced relative to the original dataset and clustered close to zero. Notably, the surrogate data did not reproduce the systematic Beginning vs. End increase observed in the empirical results (mean correlation coefficients averaged across all frequency bands; Respiration‐LegL: 0.04 ± 0.06 vs. 0.009 ± 0.05; Respiration‐LegR: −0.007 ± 0.06 vs. 0.04 ± 0.08; Respiration‐BackL: 0.05 ± 0.03 vs. 0.01 ± 0.04; Respiration‐BackR: 0.009 ± 0.04 vs. 0.06 ± 0.05; all *p* > 0.05). The surrogate data did not reproduce the fatigue‐dependent increase observed in the original results, suggesting that preserved spectral structure and trends alone are insufficient to account for the observed coupling.

In a purely common‐driver scenario in which BR and EMG scale independently but monotonically with increasing workload, phase randomization of the EMG signal would preserve the global trend and overall spectral structure, and therefore would not be expected to eliminate the fatigue‐dependent increase in cross‐correlations. The fact that surrogate destruction of temporal organization eliminates this effect suggests that preserved temporal coordination may contribute to the observed coupling, beyond simple parallel scaling driven by the trend. Together, the detrended and surrogate results suggest that respiratory‐muscular coupling during incremental maximal cycling reflects two complementary components: (i) a dominant large‐scale coordinated scaling associated with graded workload and fatigue progression; and (ii) a modest residual short‐timescale coupling that is embedded in the trend and persists after trend removal.

Finally, to assess the statistical baseline and interpretational reference of the observed network organization and its changes with fatigue, we introduced an additional step in our surrogate test to determine the significance threshold of this dataset for network link strength. We found that the significance threshold for network link strength was Th_exercise_ = 0.2 (corresponding to the highest Th value during the exercise segments). This threshold is now represented as a black horizontal line in the bar charts (Figures 3 and 4).

## 4 DISCUSSION

This study investigated how respiratory dynamics coordinates with the activity of distinct muscles during two consecutive cycling tests performed until exhaustion, and examined how the respiratory‐muscular network reorganizes with fatigue. We utilize the amplitude‐amplitude cross‐frequency coupling (ACFC) that quantifies the temporal co‐modulation between fluctuations in breathing rate and EMG spectral power across frequency bands and muscles. In summary, we report: (Kenney et al., 2015) We provide the first quantitative characterization of respiratory‐muscular coupling at the network level, revealing a complex hierarchical organization of link strength that reflects the functional role of each muscle during cycling; (Sharples et al., 2022) a marked increase in respiratory‐muscular coupling as fatigue accumulates within Exercise 1, consistent with the growing metabolic and ventilatory demands of the test; and (Hall, 2016) a slight reduction in respiratory‐muscular coupling when comparing Exercise 2 to Exercise 1, likely reflecting the influence of residual fatigue on the respiratory‐muscular network.

To better interpret the findings of this study, it is important to highlight some key technical aspects of the analyzed signals. The BR time series contains two main components: its level, which reflects the instantaneous BR, and its amplitude, which reflects the variability of this rate over time. On the other hand, the EMG frequency‐band time series represents the temporal dynamics of the spectral power of muscle activation within a specific frequency range. In this context, the power level of the EMG frequency‐band time series corresponds to the characteristic intensity of motor‐unit activity within that band, while the amplitude reflects the variability in the power of this activation over time.

Additionally, in the context of the incremental cycling protocol used here, both BR and EMG spectral power time series exhibit a pronounced monotonic increase reflecting progressive workload and fatigue accumulation. This slow incremental trend carries physiologically meaningful information regarding system‐level adaptation to exercise demands (Methods Section 2.4.5). Accordingly, respiratory‐muscular coupling in this paradigm can be interpreted at two complementary levels: (i) a dominant large‐scale coordinated scaling associated with graded workload and fatigue progression; and (ii) a modest residual short‐timescale coupling that is embedded in the trend and persists after trend removal.

### 4.1 Respiratory‐muscular networks during exercise 1: Beginning versus end

At the Beginning of Exercise 1, all respiration‐muscle sub‐networks exhibit weak link strength across EMG frequency bands (Figure 3). At this stage, metabolic and ventilatory demands are relatively low, and respiratory dynamics display greater temporal variability compared to later phases of the exercise test (Beginning panels in Figure 2b). Under these conditions, fluctuations in BR and EMG frequency bands time series show limited amplitude co‐modulation, resulting in low cross‐correlation coefficients C. During this initial phase, the vastus lateralis primarily generates the force required for pedaling, while the erector spinae contributes mainly to trunk stabilization, with comparatively lower overall activation. Ventilatory adjustments during low‐intensity exercise arise from integrated neural and peripheral influences operating under modest physiological demand (Boone et al., 2016; Forster et al., 2012; Kenney et al., 2015). Within this context, the reduced co‐modulation between respiratory and neuromuscular signals may account for the weak respiratory‐muscular coupling observed at the Beginning of Exercise 1, manifested for all network links.

As exercise intensity rises and fatigue accumulates at the End of Exercise 1, respiratory‐muscular coupling increased across all sub‐networks involving different muscles. From a signal perspective, BR exhibits a clear progressive increase over time (Figure 2b). Likewise, EMG frequency‐band time series display a progressive increase in power with exercise effort. Together, the simultaneous rise in respiratory dynamics and neuromuscular activation results in stronger amplitude co‐modulation between BR and EMG spectral power time series, yielding higher *C* values at the End of Exercise 1. From a physiological perspective, the growing metabolic demand imposed by the cycling test requires a sharp increase in oxygen uptake and carbon dioxide elimination (Beaver et al., 1986; Binder et al., 2008; Skinner & Mclellan, 1980). Although metabolic demand contributes to ventilatory adjustments, breathing frequency does not necessarily scale proportionally with V̇O_2_ or V̇CO_2_, particularly at higher intensities (Forster et al., 2012; Thompson et al., 2025). At the same time, overall myoelectrical activity increases with higher force output requirement (i.e., gradual watts increment) and fatigue accumulation, reflecting greater motor‐unit recruitment (Beck et al., 2014; Henneman et al., 1965; Mendell, 2005; Wakeling, 2009). The convergence of central and peripheral influences during sustained workload may contribute to the more synchronous amplitude co‐modulation over time observed between BR and EMG frequency bands. Together, these adaptations suggest that, near exhaustion, respiration and muscle activity exhibit stronger system‐level integration to support performance under high physiological stress.

Interestingly, the shape of the link strength profiles differs comparing the Respiration‐Leg to the Respiration‐Back sub‐networks. At the end of Exercise 1, the weakest link strength in the Respiration‐Leg sub‐networks was found in the (10–30 Hz) band, whereas the strongest coupling was in (30–50 Hz) (Figure 3b). The weaker coupling likely reflects the slower, more peripheral components of the EMG signal, such as low‐frequency force fluctuations, postural adjustments, or movement‐related oscillations, which are less directly influenced by central ventilatory control. In contrast, the stronger link strength observed is consistent with frequency ranges previously associated with common corticospinal inputs in inter‐muscular coherence studies. Prior work has shown that coherence between muscles often peaks between 25 and 45 Hz, a range linked to descending cortical and subcortical drive to spinal motor neurons (Conway et al., 1995; de Vries et al., 2016; Farmer et al., 1993; Kattla & Lowery, 2010; Kerkman et al., 2018). While the present ACFC method does not allow direct inference about neural generators, the frequency‐specific stratification pattern observed for network link strength (Figure 3b) is consistent with the possibility that shared supraspinal modulation contributes to Respiration‐Leg coupling during high‐intensity exercise.

Regarding the Respiration–Back subnetworks, link strength was weak for [F1–F2] but became broadly strong across higher frequencies [F3–F10] (65–225 Hz). The erector spinae functions as a tonic stabilizer during cycling, with motor units active almost continuously to maintain trunk posture. This sustained activation may cause extensive temporal overlap of MUAPs, broadening the EMG spectrum into higher frequencies (without implying that individual motor units fire at such high rates). Although respiration occurs at a much slower rhythm, each breathing cycle alters thoraco‐abdominal pressure and trunk mechanics, producing small but consistent modulations in the overall EMG amplitude of erector spinae. These slow amplitude modulations affect all frequency components of the EMG simultaneously, since they scale the entire signal up and down. Because ACFC method captures correlations between the amplitude envelopes of BR and each EMG frequency band, such global amplitude modulation produces coherent fluctuations across both low‐ and high‐frequency bands—yielding broadband respiratory–muscular coupling. This reasoning may be further supported by the use of 20‐s windows to compute EMG spectral power (see Methods), which smooth the signal and emphasize slow fluctuations in the signal. In contrast, the vastus lateralis contracts rhythmically with pedaling (with alternation between activation and relaxation); here, respiratory modulation aligns primarily with the dominant rhythmic component around 30–50 Hz [F2], yielding a narrower frequency‐specific coupling.

The increased degree of respiratory–muscular coupling with fatigue (i.e., Beginning vs. End) observed in this study contrasts with previous findings on cardio‐muscular coupling, which reported decreased coupling as fatigue accumulated (Garcia‐Retortillo & Ivanov, 2025). This discrepancy likely reflects differences in the physiological demands of the exercise protocols. The present study used a cycling test characterized by a strong cardiorespiratory load, whereas the previous study employed a squat test imposing primarily peripheral muscular demand (Garcia‐Retortillo & Ivanov, 2025). Accordingly, the higher respiratory‐muscular coupling observed here likely reflects the dominant role of ventilatory regulation under high cardiorespiratory stress, while the reduction in cardio‐muscular coupling during the squat test may indicate that, under predominantly peripheral fatigue, neural and metabolic resources are redistributed toward the working muscles. Together, these findings highlight the sensitivity of the ACFC method to detect task‐specific patterns of multi‐system coordination.

### 4.2 Respiratory‐muscular networks: Exercise 1 versus exercise 2

When comparing Exercise 2 to Exercise 1, a small, non‐significant reduction in link strength was observed across all respiratory‐muscular sub‐networks (Figure 4). This slight decline likely reflects the residual effects of fatigue generated during Exercise 1, suggesting that the 5‐min rest between sets provided partial but incomplete recovery of respiratory‐muscular coupling. Although the network appears to regain most of its link strength during the recovery period, it does not fully return to the baseline level observed for Exercise 1. A more pronounced reduction in link strength would likely emerge after multiple consecutive bouts, when the capacity for full cardiorespiratory and neuromuscular recovery becomes more limited.

Regarding the shape of the links strength profile, the strength of the links remained relatively consistent across frequency bands and across all respiratory‐muscular sub‐networks (Figure 4). Unlike the comparison between the Beginning and End of Exercise 1 (Figure 3)—where a hierarchical organization of links strength was evident—the comparison between Exercises 1 and 2 revealed a more uniform profile (Figure 4b). This indicates that residual fatigue exerts a broad, mild dampening effect on respiratory‐muscular coupling, rather than altering specific frequency bands. These results emphasize the value of examining respiratory‐muscular coupling over shorter timescales, as the fine temporal reorganization captured between the Beginning and End of Exercise 1 is less evident when averaging across entire exercise bouts.

### 4.3 Practical applications

This work represents an initial step toward the development of a new class of network‐based markers that quantify how distinct physiological systems coordinate during exercise. Together with our previously developed inter‐muscular (Garcia‐Retortillo, Abenza, Vasileva, et al., 2025; Garcia‐Retortillo et al., 2023; Garcia‐Retortillo & Ivanov, 2022) and cardio‐muscular coordination (Garcia‐Retortillo & Ivanov, 2025) markers using the ACFC method, the present respiratory‐muscular analysis presented here extends this framework by providing additional insight into multi‐system coordination. The proposed new network‐based markers could provide complementary information to conventional physiological measures such as VO_2_max, ventilatory thresholds, heart rate, and power output that reflect performance of individual systems. In contrast, markers derived from dynamic networks offer a framework to quantify how systems coordinate as an integrated adaptive network to sustain function. The reported findings should be interpreted as exploratory, proof‐of‐concept evidence that respiratory‐muscular coupling can be quantified dynamically during incremental exercise.

### 4.4 Limitations

This study represents an initial step toward quantifying respiratory‐muscular coupling during incremental exercise, and several limitations should be acknowledged. First, ACFC quantifies functional amplitude coupling between BR dynamics and EMG spectral power time series but does not identify specific neural mechanisms underlying this coordination. Contributions from cortical motor areas, brainstem respiratory centers, and group III/IV muscle afferents cannot be disentangled using sEMG and respiration belt recordings alone (Amann et al., 2010; Schottelkotte & Crone, 2022). Future studies combining electrophysiological signals with other neural and physiological recordings (e.g., neuroimaging, fNIRS, metabolic breath‐by breath data) are warranted. Second, the accumulation of fatigue induced by the incremental protocol inherently imposes slow trends in the recorded signals. Further studies using distinct methodologies are required to investigate the microarchitecture of sEMG dynamics within a breathing cycle as a function of fatigue. Third, only a limited number of muscles were analyzed, which do not fully capture the spatial complexity of respiratory‐muscular interactions across the body. Future studies should include additional muscles to build a more comprehensive network representation. Fourth, sEMG is inherently affected by factors such as tissue filtering, cross‐talk, and electrode placement, which may influence spectral estimates (Farina & Rainoldi, 1999; Germer et al., 2021; Hermens et al., 2000; Merletti & Muceli, 2019). Expanding this framework to distinct movement and protocols, larger and more diverse populations, and complementary physiological measures could strengthen future work and advance the understanding of the human organism as an adaptive, dynamic network.

## 5 CONCLUSIONS

In summary, our findings report: (Kenney et al., 2015) the first quantitative characterization of respiratory‐muscular interaction networks, revealing a complex hierarchical organization of link strength that reflects the functional role of distinct muscles during cycling; (Sharples et al., 2022) a significant increase in the strength of respiratory‐muscular network links as fatigue accumulates during Exercise 1, reflecting the growing metabolic demands with progression of the exercise test; (Hall, 2016) slight reduction in respiratory‐muscular interactions when comparing Exercise 2 to Exercise 1, quantifying the influence of residual fatigue on the respiratory‐muscular network; and (McArdle et al., 2015) increased heterogeneity in the respiratory‐muscular network links strength with the accumulation of fatigue. These network‐level adaptations demonstrate that physiological responses to exercise arise not only from isolated systems, but also from their dynamic interaction as an integrated network. Respiratory‐muscular coupling, alongside other network‐based markers, may help extend the assessment of human function beyond individual system performance toward an adaptive, dynamic network perspective.

## AUTHOR CONTRIBUTIONS

**Sergi Garcia‐Retortillo:** Conceptualization; data curation; funding acquisition; investigation; methodology; supervision. **Javier O. Pinzon Arenas:** Formal analysis; investigation; methodology; visualization. **Óscar Abenza:** Formal analysis; investigation; methodology; visualization. **Hugo F. Posada‐Quintero:** Data curation; formal analysis; methodology; software; supervision. **Plamen Ch. Ivanov:** Conceptualization; methodology; supervision; validation.

## FUNDING INFORMATION

We acknowledge support from the Translational Research Center (Wake Forest University; ID0576‐590006‐U05244), and from the W.M. Keck Foundation.

## CONFLICT OF INTEREST STATEMENT

The authors declare no conflict of interest. Generative AI was used during the preparation of this manuscript. Specifically, ChatGPT Plus using GPT‐4o (OpenAI) was used in 2025–2026 to assist with language editing, refinement of sentence structure, and improvement of readability. Its use was limited to language clarity and grammar, and was applied to the Abstract, Introduction, Methods, Discussion, Limitations, and Conclusions. No generative AI tool was used to produce, analyze, or interpret experimental data, perform statistical analyses, or create figures. All AI‐assisted text was reviewed, edited, and approved by the authors. The authors take full responsibility for the accuracy, integrity, and originality of the manuscript content. To the best of the authors' knowledge, the AI technology used does not retain copyright over the generated work product in a manner that would prevent publication.

## Data Availability

All data supporting the findings of the present study are presented in the article. However, any additional data are available upon a reasonable request from the corresponding author.
